# On-Column Preparation of High-Performance Affinity Chromatography Columns

**DOI:** 10.1021/acs.analchem.6c01884

**Published:** 2026-07-23

**Authors:** Martin Gilar, Beatrice Muriithi, Yeliz T. Sarisozen, Philip Wuthrich, Michael Donegan, Kevin D. Wyndham

**Affiliations:** 36565 Waters Corporation, Milford, Massachusetts 01757-3696, United States

## Abstract

We describe a custom preparation of high-performance affinity columns based on 3.5 μm nonporous polymeric particles covalently modified with streptavidin. The sorbent was packed into a 2.1 × 20 mm column hardware compatible with pressures up to 10,000 psi (68 MPa). Biotinylated affinity ligands were then immobilized on-column using a repetitive injection method until the streptavidin sorbent was saturated. The saturation was observed as a ligand breakthrough signal by ultraviolet detection. Column binding capacity of immobilized affinity ligands was estimated for d-biotin, biotinylated 25nt oligodeoxythymidine, and biotinylated anti-insulin mAb ligands. The prepared columns were used to quantify mRNA and insulin. Affinity column performance was evaluated in terms of binding capacity, calibration linearity range, and target analyte limit of quantitation. The high-performance streptavidin column is a platform for custom-made affinity chromatography device preparation, enabling routine quantitation of analytes of interest.

## Graphical abstract

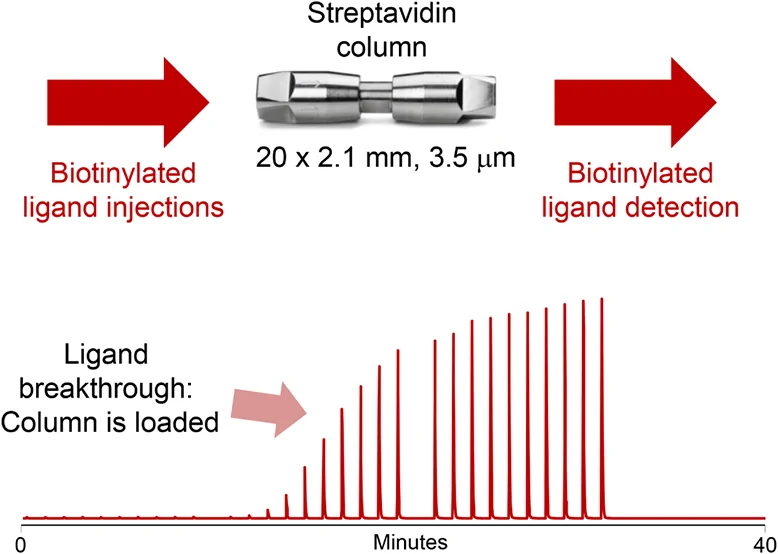

Affinity liquid chromatography (aLC) is a chromatographic method that utilizes selective interactions between immobilized affinity ligands and the analyte of interest or a class of target analytes.^1,2^ A common example of aLC is the selective purification of immunoglobulins (IgG) using Protein A or Protein G ligands immobilized on solid supports.^3^ Protein A columns are widely used for the purification of therapeutic monoclonal antibody (mAb) drugs.^4^ Other applications of aLC are the isolation of low-abundance proteins,^5^ glycoproteins,^6,7^ histidine-tagged proteins, and mRNA.^8,9^

In contrast to other chromatographic modes, which are capable of resolving multiple analytes within a single analysis, aLC in principle produces two fractions: the unretained fraction containing sample constituents that have minimal interaction with the immobilized affinity ligand and a second fraction containing the target analyte that is eluted from the column with a (step) gradient of a mobile phase.^10^ aLC is often performed with a plastic (bioinert) or glass column housing under low-pressure LC conditions.^11^ aLC columns are available in various formats, including sorbent-packed beds, monoliths, or membrane devices. The applications of aLC were recently reviewed by Bilkova et al.^12^

aLC columns compatible with LC pressures are available for selected applications, such as Protein A columns for monitoring mAb production (titer measurement),^13,14^ oligodeoxythymidine columns for quantification of mRNA^15–18^ or antibody-based columns for purification and analysis of adeno-associated viruses (AAVs).^19–21^

Development of affinity chromatography methods for custom applications requires the development of custom affinity ligands, selection of a suitable sorbent, immobilization of the affinity ligands onto the sorbent, and packing of the affinity sorbent into column hardware.^1^ This process requires mastery of multiple specialized skills, which makes the development of new affinity chromatography devices difficult. A possible solution is to use a generic immobilization method that enables the chemical reaction of an affinity ligand with an activated sorbent. Aldehyde, amino, azide, and other functional sorbent chemistry groups were proposed for biomolecule immobilization on solid supports.^22^ The formation of a noncovalent streptavidin–biotin complex is also used to immobilize biotinylated molecules on streptavidin-modified solid supports.^12^ The biotin–streptavidin bond is highly stable across a wide range of pH levels and mobile phases containing high proportions of organic solvents.^23,24^

In a recent report, we described the preparation of a Protein A affinity column by directly immobilizing Protein A on a hydrophilic surface-modified polystyrene-divinylbenzene (PS-DVB) nonporous sorbent.^25^ The 2.1 × 20 mm column packed with 3.5 μm Protein A particles exhibited high performance for mAb quantitation. The column was suitable for fast and sensitive mAb titer analysis.^25^ Unlike the majority of the aLC columns on the market, the chosen column hardware is compatible with high pressure (10,000 psi), enabling high-throughput analyses using fast flow rate,^25^ or direct column coupling with a second LC dimension, e.g., a size-exclusion (SEC) column for mAb analysis.^26^

In the present work, we describe the application of the same hydrophilic PS-DVB sorbent with immobilized streptavidin. The streptavidin column platform was used for custom immobilization of biotinylated affinity ligands via the biotin–streptavidin complex.^27,28^ In this work, we describe a novel on-column efficient preparation of affinity columns with biotinylated oligodeoxythymidine (dT25) and biotinylated anti-insulin mAb (Ins mAb). The first goal was to demonstrate the on-column ligand immobilization using serial injections of small amounts of biotinylated ligands.^29^ The second goal was to estimate the maximum binding capacity of the streptavidin column using d-biotin and compare it with the binding capacity for biotinylated ligands, such as biotinylated oligodeoxythymidine and biotinylated anti-insulin mAb. The third goal was to illustrate the utility of the prepared affinity columns, develop methods for mRNA and insulin analysis, and characterize column performance in terms of limits of binding capacity, calibration linearity, and sample carryover. The overall goal was to describe the preparation and utility of aLC applicable in the standard analytical core lab and demonstrate the novel tandem dT25 × SEC method for the analysis of mRNA.

## Experimental Section

### Chemicals and Samples

Sodium phosphate monobasic (≥99.0%, catalog no. S8282), sodium phosphate dibasic (≥99.0%, catalog no. S7907), and ortho-Phosphoric acid (85%, catalog no. 345245) were purchased from Sigma-Aldrich (St. Louis, MO, USA). Fisher d-biotin (catalog no. BP232-1) and Micro BCA assay kit (CAS 23235) were purchased from Thermo Fisher Scientific (Waltham, MA, USA). All reagents were used as purchased, without further purification. All buffers were prepared with Milli-Q water and filtered through a 0.2 μm filter (catalog no. FB12566508) purchased from Fisher Scientific (Hampton, NH, USA). 5′-Biotinylated dT25 oligodeoxythymidine (25 nucleotides long, HPLC purified) and dA20 oligodeoxyadenosine (20 nucleotides long, crude) were purchased from Integrated DNA Technologies (Coralville, IA, USA). N1-methyl-pseudouridine modified mRNA samples (859 nt, M6 EPO mRNA, part no. L-8109; 997 nt, M6 EGFP mRNA, part no. L-8101; 997 nt, M6 Cherry mRNA, part no. L-8103; 1351 nt, M6 Cre mRNA, part no. L-8111; 1922 nt, M6 Fluc mRNA, part no. L-8102; and 4522 nt, M6 Cas9 mRNA, part no. L-8106) were purchased from TriLink BioTechnologies (San Diego, CA, USA). Tail-less 2180 nt mRNA was obtained from Kactus Bio, (Waltham, MA, USA). Biotinylated insulin antibody (Ins mAb, part no. 61-1054) was purchased from Biosynth International, Inc. (Gardner, MA, USA). Human insulin (catalog no. PHR8925) was purchased from Sigma-Aldrich (St. Louis, MO, USA).

### Chromatographic Columns and Instruments

The 3.5 μm nonporous polystyrene/DVB particles were prepared, hydrophilic surface-modified, and then conjugated with streptavidin by the Waters R&D and manufacturing teams.^25,30^ The amount of conjugated streptavidin on the sorbent was measured by the Micro BCA Assay kit (CAS 23235) purchased from Thermo Fisher Scientific (Waltham, MA) and used according to the manufacturer’s protocol. The measured streptavidin amount was 6.7 μg/mg of sorbent (batch YTS-11-172). The sorbent was packed in-house using Waters MaxPeak Premier Column Hardware 2.1 × 20 mm from Waters Corporation (Milford, MA, USA). The mass of sorbent packed into the column (47.9 ± 1.3 mg; RSD 2.6%) was measured by emptying the packed column and weighing the dry particles (n = 3). Chromatographic experiments were performed using the ACQUITY Premier System consisting of a quaternary solvent manager (QSM), Flow Through Needle Sample Manager (FTN SM), column heater manager (CM-A) with an enabled active preheater (APH), and an ACQUITY Photodiode Array (PDA) Ultraviolet Detector with a 5 mm titanium flow cell. Data were collected and processed with Empower software from Waters Corporation (Milford, MA).

### Preparation of Affinity Column: Biotinylated Ligands Bonding on Streptavidin Column

The streptavidin column was equilibrated with 0.1 M sodium phosphate binding buffer, pH 7.4, for approximately 20 column volumes (4 min at 0.15 mL/min; the buffer was prepared by dissolving 1.14 g of sodium phosphate monobasic and 5.75 g of sodium phosphate dibasic in 0.5 L of water). Biotinylated affinity ligands were immobilized on a streptavidin column using a UPLC system and repeated injections of biotinylated molecules dissolved in the 0.1 M phosphate binding buffer. The injections of biotinylated ligands were typically repeated at 1 min intervals until breakthrough was observed by UV detection, indicating column saturation. We used the Multiple Injections in a Single Experimental Run (MISER) method to load the column with affinity ligands and monitor breakthrough.^29,31,32^ The loading was performed at a 0.15 mL/min flow rate of the binding buffer and 25 °C. The PDA detector sampling rate for the MISER experiment was 40 points/sec, with the fast filter setting. Examples of MISER column preparation are shown in Figure 1 and described in Figure 1 captions. Biotinylated dT25 ligand was immobilized on a streptavidin column using 2 μL injections of 0.25 nmol/μL ligand solution. The Ins mAb column was prepared by injecting 6 μL injections of 8.87 pmol/μL biotinylated anti-insulin antibody ligand solution (Figure 1). Two columns of each ligand type were prepared to evaluate the repeatability of column bonding. The column binding capacity was nearly identical in repeated experiments, as shown in Supporting Information Figure S1 and Figure S2.

**Figure 1:**
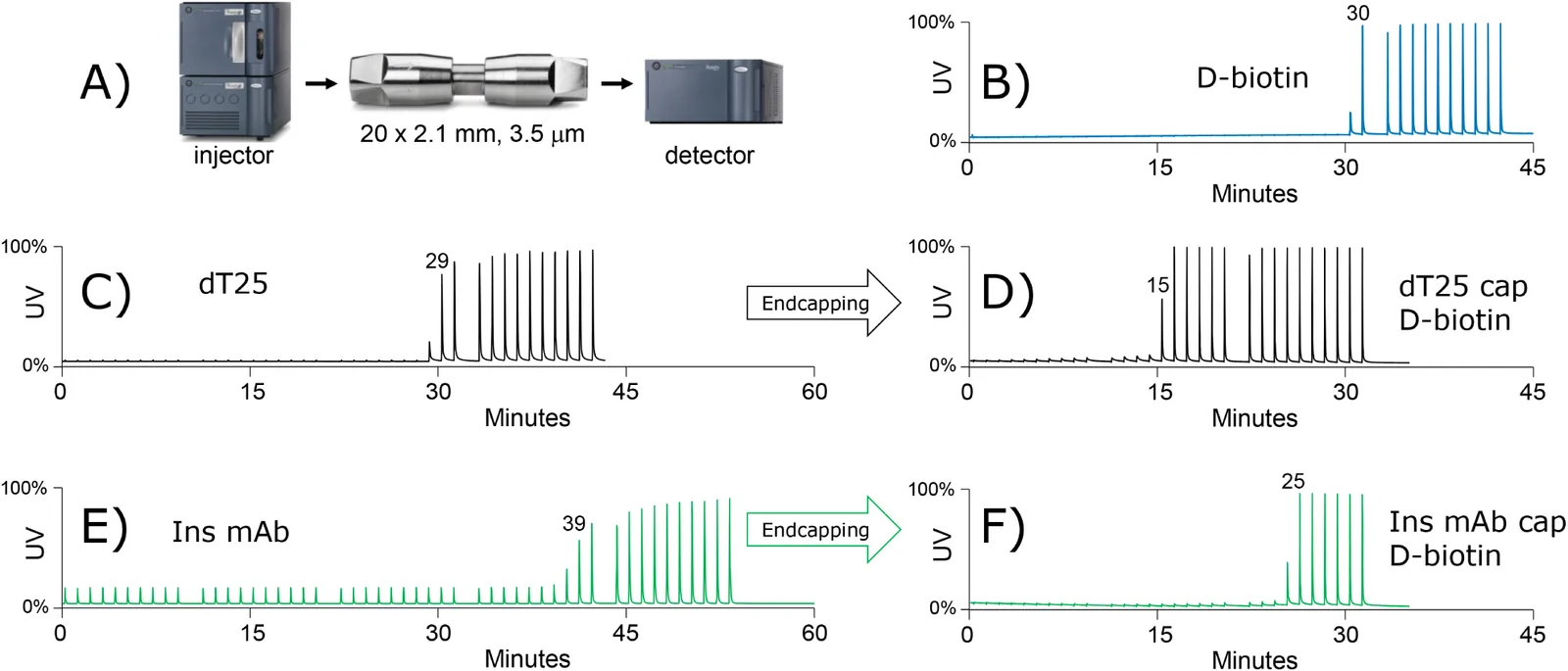
Examples of on-column aLC device preparation with repetitive injections of biotinylated ligands. A) A streptavidin column is placed between the injector and detector. B) Evaluation of the streptavidin column loading capacity with 1 μL of 1 nmol/μL d-biotin injections. C) Fresh streptavidin column loading with biotinylated dT25 ligand; 0.25 nmol/μL, 2 μL injections. D) End-capping of dT25 column with an additional 1 μL of 1 nmol/μL d-biotin injections. E) Streptavidin column loading with biotinylated Ins mAb; 8.87 pmol/μL, 6 μL injections. F) Ins mAb column end-capping with 1 μL of 1 nmol/μL d-biotin injections.

After the immobilization of the biotinylated ligand, the remaining streptavidin unbound sites (presumably due to steric hindrances) were capped by a second MISER experiment injecting a 1 nmol/μL solution of free biotin dissolved in the binding buffer. This so-called end-capping procedure was implemented to ensure that all streptavidin active sites on the column were biotinylated and that no other biotinylated ligands could be inadvertently immobilized on the column throughout its use. The same end-capping procedure, injecting a 1 nmol/μL biotin solution at a 0.15 mL/min flow rate of the binding buffer, was utilized to estimate the maximum streptavidin column binding capacity (for biotin).

### Affinity Chromatography Methods

The prepared affinity columns were used to selectively retain and quantify selected target analytes. The dT25 column was used for quantitation of mRNA (hybridization of the polyA tail of mRNA with the immobilized dT25 ligand) as follows: the dT25 column was equilibrated with binding buffer A (0.1 M sodium phosphate, pH 7.4) at 0.4 mL/min and 25 °C. The mRNA sample prepared in binding buffer A was injected and trapped on the column. mRNA was eluted with mobile phase B (10% acetonitrile in water). A step gradient from 100% A to 100% B was executed at 0.01 min, held at 100% B for 2.5 min, and then switched back to 100% A and held until 6 min for full column equilibration prior to the next analysis. This method was suitable for mRNA samples and dA20. UV detection was performed at 260 nm unless stated otherwise.

An insulin affinity column was used for the quantification of human insulin. Binding buffer A was 0.1 M sodium phosphate, pH 7.4, and elution buffer B was a 0.2% phosphoric acid solution. Significant breakthrough was observed when loading insulin at a flow rate of 0.4 mL/min. Therefore, sample loading was performed at 0.04 mL/min for 2 min; between 2 and 2.1 min, the mobile phase flow rate was ramped up to 0.4 mL/min, and the mobile phase composition was switched from 100% A to 100% B to elute the captured insulin. The column temperature was at 25 °C. UV detection was performed at 280 nm.

The dT25 × SEC tandem column method for mRNA analysis (polyA-tailed vs tailless mRNA quantitation) used a 2.1 × 20 mm dT25 column serially connected to a GTxResolve Premier SEC 1000 Å, 3 μm, 4.6 × 300 mm column. The tandem columns were equilibrated with 0.1 M of sodium phosphate, pH 7.4, at 0.4 mL/min and 25 °C. 2.5 μg of mRNA sample was injected onto the dT25 column. The unretained tailless mRNA fraction is resolved by SEC column with the same isocratic sodium phosphate mobile phase. After 12 min, a 25 μL injection of pure water was used to elute the dT25 bound polyA-tailed mRNA fraction, and the second SEC analysis was performed with the sodium phosphate buffer as the eluent.

## Results and Discussion

### On-Column Preparation of Affinity Columns

The 2.1 × 20 mm streptavidin columns were connected to an HPLC instrument and equilibrated with the binding mobile phase A (0.1 M sodium phosphate, pH 7.4) at 0.15 mL/min. An HPLC system with an FTN Sample Manager was used to load a defined number of small-volume injections of the biotinylated sample onto the column. Column loading with biotinylated ligands was performed in the MISER mode under continuous UV monitoring. Examples of MISER chromatograms illustrating the preparation of the two most common types of aLC columns (i) based on immobilized nucleic acid (dT25) or (ii) antibody (anti-insulin mAb) are shown in Figure 1. The MISER mode permits convenient detection of column saturation with the biotinylated ligand; above the saturation point, the injected biotinylated ligand breaks through the column and appears as a peak(s) in the chromatogram. The peak areas were easily quantified. In Figure 1, we report the percentage of breakthrough normalized to the 100% area of the ligand (measured in a separate injection of the sample with no column in the flow path).

MISER loading of the streptavidin column, as described in Figure 1, is comparable to feeding the column with a continuous feed of biotinylated ligand, with several advantages: (i) small discrete injections of biotinylated ligands enable convenient monitoring of the breakthrough. The loading can be stopped immediately upon breakthrough detection, thereby saving an expensive biotinylated ligand. The efficiency of on-column affinity LC column preparation is the primary motivation for the MISER approach. (ii) One can avoid pump flow path contamination with the biotinylated ligands. (iii) Column loading with MISER can be easily shortened by using larger sample injection volumes in the subsequent column preparation (see Supporting Information Figure S2).

Figure 1 illustrates the measurement of streptavidin column capacity. The MISER experiment was performed in a series of 10 injections with a 1 min gap programmed between series for clearer visual data interpretation. We first use 1 μL injections of 1 nmol/μL biotin samples (Figure 1B) to estimate the full column binding capacity, assuming that biotin, a small molecule, binds to the immobilized streptavidin without steric hindrances. We observed 52.5% breakthrough of biotin at the 29th injection and a complete breakthrough (100.2%) at the 30th injection (biotin peaks were detected at UV 210 nm). Similar results were reproduced for a second column (data not shown). We concluded that the 20 × 2.1 mm streptavidin column binding capacity for biotin was between 29 and 30 nmol. Please note that after this capacity test, the column was fully saturated with biotin and cannot be used for further biotinylated ligand immobilization.

In further experiments, we used fresh streptavidin columns to prepare two different types of affinity columns. Figure 1C illustrates the immobilization of dT25 biotinylated oligonucleotide; Figure 1E illustrates the immobilization of biotinylated Ins mAb ligand. In Figure 1C, we observed 23% dT25 oligonucleotide breakthrough at the 28th injection and 60% breakthrough for injection 29 (UV 260 nm). A breakthrough rate below 0.7% was observed for injections 1–27. This “breakthrough” was due to minor sample contamination with oligonucleotides not coupled with biotin (failed synthesis products). The experiment in Figure 1E shows a more prominent but constant level of signal in early injections for Ins mAb (9.5%). This breakthrough is presumably due to a fraction of Ins mAb not conjugated with biotin, and therefore not immobilized on the streptavidin column, rather than the breakthrough of the biotinylated ligand or UV-absorbing sample constituents (Supporting Information Figure S3). The peak numbers highlighted in Figure 1 represent the injection during which the breakthrough of ligand exceeded 50%. The breakthrough of Ins mAb occurred in the 39th injection column preparation (Figure 1E).

The mass balance of ligands immobilized on streptavidin columns is presented in Table 1; the ligand amount is calculated from the number of injections, sample volume, and sample concentration. The immobilized amounts were corrected for the fraction of nonbiotinylated ligand (molecules unbound to the column due to the absence of a biotin moiety, as mentioned earlier). Table 1 reveals that the amount of immobilized ligand (nanomoles) decreases with its molecular weight. This observation suggests that steric hindrance limits the molar amount of ligands that can be coupled to streptavidin columns. Presumably, a fraction of streptavidin binding sites cannot be accessed by bulky biotinylated ligands because of steric hindrance and may remain uncoupled.

**Table 1:** Mass Balance of Immobilized Biotinylated Ligands on Streptavidin Columns

Ligand type	Ligand immobilized (nmol/column)	Biotin end-capping (nmol/column)	# Biotins/ligand	Total binding capacity (nmol)
Biotin	29	-	1	29
dT25	14	15	1	29
Ins mAb	1.7	24	∼3	∼29

To verify this hypothesis, we performed a second experiment and coupled the prepared affinity columns with additional injections of a biotin solution. The end-capping experiment is presented in Figure 1D and 1F. For instance, the column that was coupled with the dT25 ligand required an additional 15 injections (15 nmol) of biotin to saturate the unbound streptavidin sites (Figure 1D). Combining 14 nmol of dT25 ligand and 15 nmol of biotin, the total binding capacity of the column was 29 nanomoles. This value is consistent with the previously estimated column capacity with biotin only (Table 1 and Figure 1B). This simple mass balance is expected for ligands such as dT25, which contain a single biotin group per molecule. As we will discuss later, the streptavidin column binding capacity calculation must consider the number of biotin groups conjugated to the immobilized affinity ligand.

Exposing aLC columns to an excess of biotin could potentially strip a portion of the immobilized biotinylated affinity ligand.^24^ Therefore, we monitored the end-capping experiment using UV at 210 nm (detection of biotin), 260 nm (detection of dT25), or 280 nm (detection of Ins mAb). The results in Supporting Information Figure S4 confirm that a small portion of the immobilized dT25 ligand was indeed released by additional biotin injections most notably near the point of aLC column saturation with biotin. From the peak areas, we estimated that only 0.5% of the immobilized ligand was cumulatively released from the dT25 column by the end-capping procedure.

The data in Figure 1 and Table 1 reveal that the mass balance of affinity ligand and biotin end-capping did not yield the expected column binding capacity of 29 nmol for the Ins mAb column. This is because the mAb ligand contains more than one biotin group (Supporting Information Figure S5). LC-MS analysis reveals that mAb contains on average three biotin molecules per molecule (Supporting Information Figure S5) with a distribution of 0–5 biotins per mAb. When the stoichiometric amounts of biotin are considered, the binding capacity of the Ins mAb affinity LC column is between 29 and 30 nmol (Table 1).

### Affinity LC Columns Applications, Limits of Quantitation, and Calibration Linearity

Affinity columns prepared in the previous section were used for the analysis of target compounds. The dT25 column was used for the quantification of mRNA that selectively hybridizes with the dT25 column by the mRNA polyA tail. Tailless mRNA does not interact with the sorbent and elutes from the column unretained (Figure 2A). The method described in the experimental section uses 0.1 M sodium phosphate as the mRNA binding buffer; alternatives such as 0.1 M ammonium acetate (pH 6.85) buffers can also be used. Elution of mRNA can be performed by applying denaturing conditions (elevated temperature, mobile phase containing denaturants, or water). We eluted mRNA with a step gradient of 10% acetonitrile in water. The proposed dT25 affinity column method was used for mRNA quantitation; the lower limit of quantitation (LLQ) with UV 260 nm detection was approximately 0.025 μg for EPO mRNA (Figure 2B). The upper range of the calibration curve linearity of 5–10 μg for EPO mRNA (Figure 2C, 2D) was not limited due to column overloading in a single mRNA injection, but rather by the upper linearity range of UV detectors (below ∼1.5 AU). The estimated carryover in subsequent blank injections was below 1% (Figure 2B).

**Figure 2:**
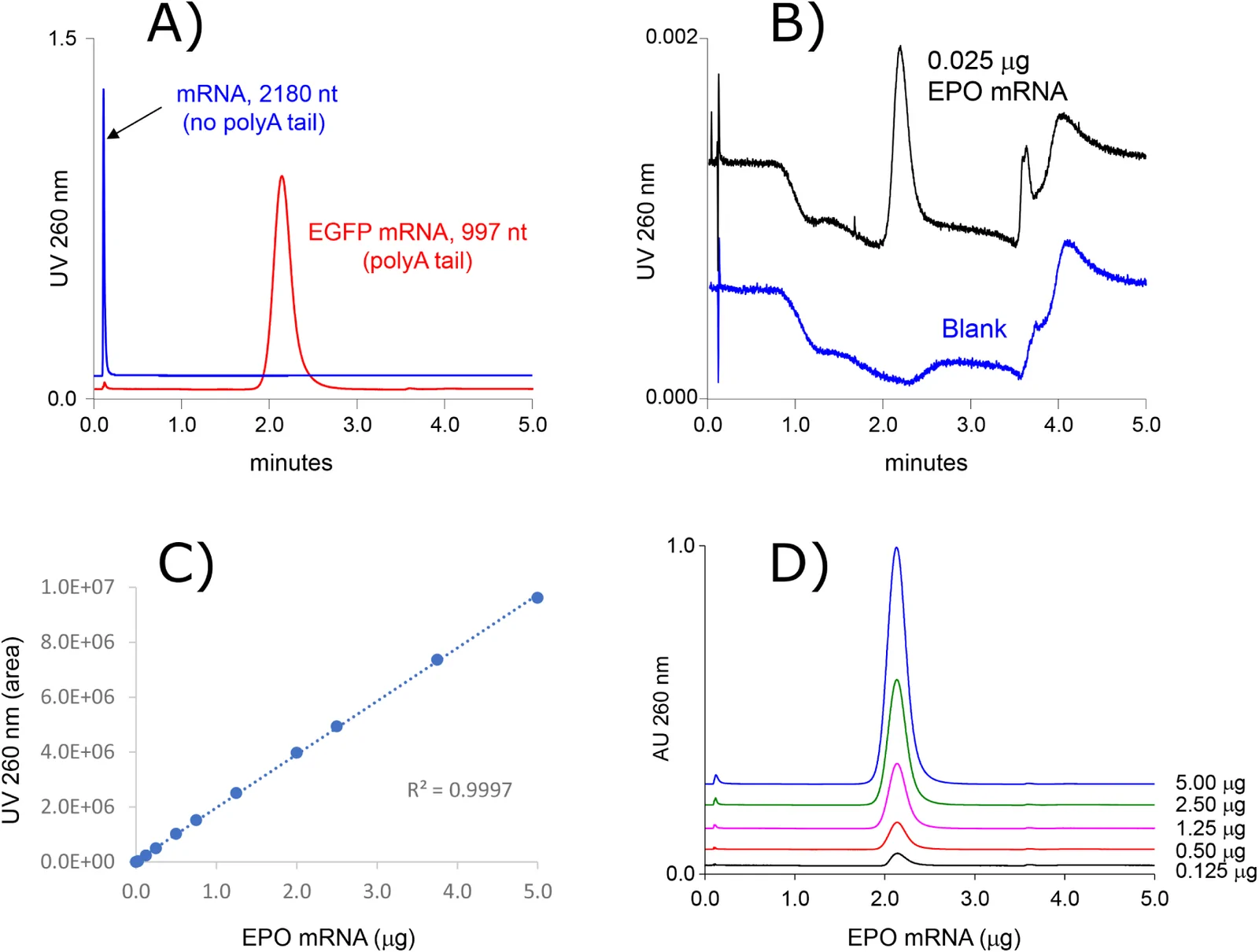
dT25 affinity LC column application to mRNA analysis. A) Selectivity with mRNA retention: polyA-tailed mRNA is retained on the column, while tailless mRNA is not. B) Limit of mRNA quantitation and blank chromatograms; mRNA binds to the dT25 column in 0.1 M sodium phosphate buffer, pH 7.4, and elution is performed with a gradient of water. C) Calibration is linear within 0.025–5 μg EPO mRNA injected on the column. D) Examples of EPO mRNA chromatograms used to construct the calibration curve.

Figure 2 shows that the background signal in a blank chromatogram is negligible. However, the initial background of a freshly prepared dT25 column was approximately 50 mAU. Before use, we implemented a wash with 5 blank gradients (see Supporting Information Figure S6A) to reduce the background. LC-MS experiments confirmed that the background signal consisted of small amounts of streptavidin and a dT25 ligand leaching from the stationary phase (Figure S6). It remains unclear whether the streptavidin–ligand complex itself is released under the operational conditions, or whether the tetrameric streptavidin complex is destabilized first, followed by monomeric streptavidin bleed (some of the streptavidin monomers are not covalently bound to sorbent). The streptavidin–dT25 complex mass was not detected; the noncovalent complex could dissipate in the ESI MS source upon ionization (Supporting Information Figure S6). For comparison, we investigated the streptavidin bleed by LC-MS for an unbound streptavidin column and observed bleed corresponding to monomeric streptavidin (Supporting Information Figure S7). While a small amount of dT25 ligand was detected by LC-MS, the bleed did not appreciably affect the dT25 column performance for 900 injections (see section Additional Considerations for aLC Columns).

Figure 3 shows examples of insulin analysis with the Ins mAb column (Figure 3A) and the calibration curve (Figure 3B). The binding buffer was 0.1 M sodium phosphate; the elution of insulin was performed with 0.2% phosphoric acid. The addition of 20% ethanol to the elution mobile phase could be used to reduce minor hydrophobic interactions of insulin with the column. Supporting Information Figure S8 shows the limits of the insulin loading capacity range. We observed sample breakthrough for a 10 μg injection on the Ins mAb column (Figure S8), which motivated the investigation of the affinity LC columns’ loading capacity for analytical applications, as described in the next section.

**Figure 3:**
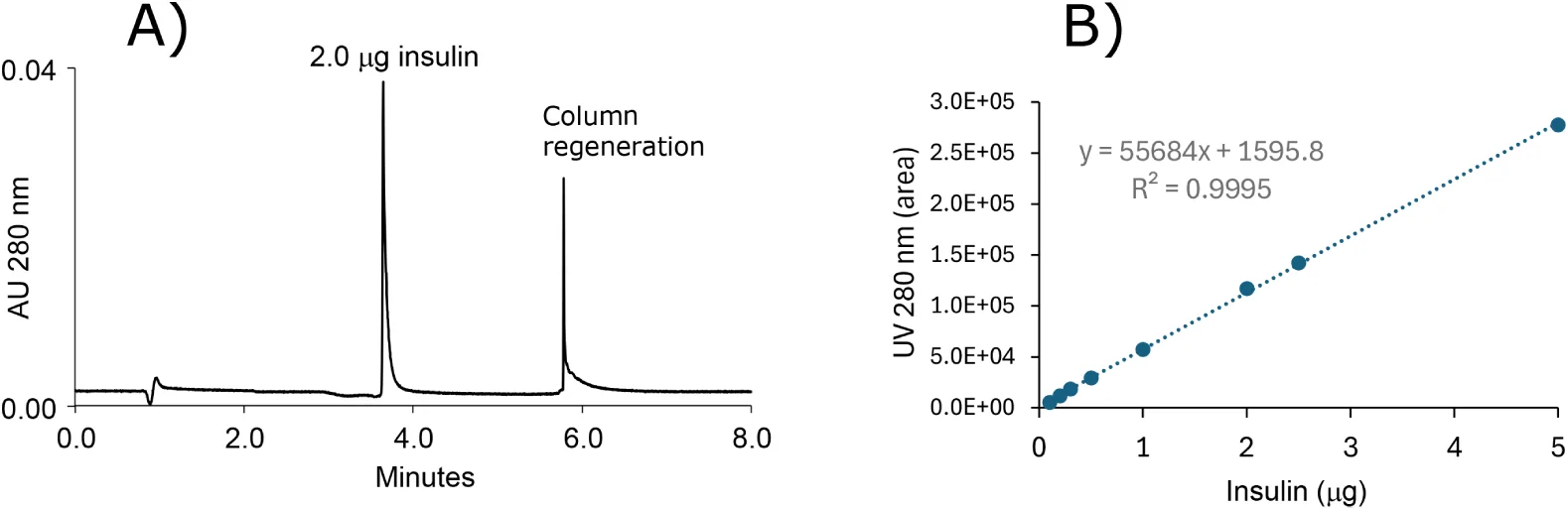
A) Ins mAb aLC column analysis of insulin. B) Calibration curve for insulin quantitation using the Ins mAb column. For aLC method details, see the Experimental Section.

### Evaluation of aLC Columns’ Binding Capacity

2.1 × 20 mm column dimensions were chosen for analytical rather than preparative applications. However, the column binding capacity does limit the upper range of the calibration curve. We measured the column binding capacity for the biotinylated ligands (Table 1). This value represents an expected maximum binding capacity for the target analyte, provided that the immobilized ligand–target molecule molar binding ratio is equimolar. For example, a dT25 column with a 14 nmol dT25 ligand capacity is expected to bind up to 14 nmol of dA20 test analyte. In practice, the dT25 column analyte capacity is limited by steric factors, especially for the bulky mRNA molecules, as discussed below.

The loading capacity of the dT25 column was measured with the MISER experiment, like the one described in Figure 1. Different mRNA samples, dA20, EGFP mRNA, or Cas9, were repeatedly injected into the dT25 column until >50% analyte breakthrough was detected, signifying the column’s dynamic binding capacity. The experiments are shown in Figures S9–S11 and summarized in Table 2. An analogous experiment was performed with an Ins mAb column using repeated injections of the insulin sample (Supporting Information Figure S12).

**Table 2:** Estimated Loading Capacity of Affinity Columns for dA20, EGFP mRNA, Cas9 mRNA, or Insulin Using Dynamic Binding Method or Static Binding Capacity Method

Ligand type	Ligand immobilized (nmol/column)	Analyte	Dynamic binding column capacity at >50% breakthrough	Static binding column capacity, elution method
dT25	14	dA_20_	9.61 nmol; 59.6 μg	6.22 nmol; 38.6 μg
dT25	14	EGFP mRNA	0.25 nmol; 81.4 μg	0.25 nmol; 81.4 μg
dT25	14	Cas9 mRNA	0.03 nmol; 45.5 μg	0.035 nmol; 55.0 μg
Ins mAb	1.7	Insulin	1.03 nmol; 6.0 μg	0.97 nmol; 5.6 μg

Table 2 reports two independent measurements of binding capacity: (i) dynamic binding capacity at the >50% breakthrough experiment and (ii) static binding column capacity obtained from the peak area of the retained analyte. The column capacity in the second experiment was estimated by eluting the analyte adsorbed on the column in the MISER experiment (after complete column saturation). The peak area of the eluted analyte was compared to that of a standard injection via union (no affinity column in the flow path). Both results for the column binding capacity are consistent. The results for the dT25 column confirm that the maximum load capacity depends on the analyte size. Oligonucleotide (dA20) mass load is comparable to the amount of dT25 immobilized ligand (9.6 nmol vs 14 nmol); for larger mRNA molecules, the mass load is significantly reduced (<0.25 nmol) and decreases with mRNA size.

Table 2 shows that for a dA20 sample, the capacity estimated with the elution experiment was 30% lower compared to the MISER binding capacity. Presumably, a portion of dA20 (and dAn-x shortmers present in the synthetic dA20 oligonucleotide) was washed from the column during its exposure to the mobile-phase flow before the elution binding capacity was performed. This effect was also observed with the Ins mAb column. Figure S12 suggests that insulin binding to the affinity column mAb is weak, and insulin is bleeding from the column during the sample load (peak tailing). When we eluted the insulin-saturated column immediately after the MISER loading experiment (Figure S12), the column capacity was consistent for both capacity methods (Table 2). However, when the saturated Ins mAb column was washed with the binding buffer (for 4 h), only 25% column capacity was observed in the static binding measurement (75% of insulin was washed away). It appeared that the Ins mAb we selected for the aLC experiment had weaker insulin binding than expected; therefore, this column was not used for further experiments.

We investigated the binding capacity of streptavidin columns using small ligands (biotin), moderately large ligands (biotinylated oligonucleotide, biotinylated nanobody), and bulky ligands, such as biotinylated mAb. The binding capacity of the streptavidin column clearly depends on the ligand size, even after taking into account the stoichiometry of biotin modifications per ligand (Table 1). The affinity column’s apparent binding capacity depends even more significantly on the analyte size, as shown in Table 2 for dA20 oligonucleotide and much bulkier mRNA samples. In other words, the development of a sorbent with a higher amount of immobilized streptavidin (6.7 μg of streptavidin per mg of sorbent was used in this study) is not likely to improve the binding capacity of the aLC column for bulky analytes such as mRNA. Large analytes may also have limited access to sorbent pores. We compared the observed binding capacity of our nonporous dT25 column with published reports; when normalized to column volume, the dT25 nonporous column capacity for mRNA was comparable to wide-pore and monolithic columns (1–2 mg of ∼4000 nt mRNA/mL of column volume).^15^

### Additional Considerations for aLC Columns

We prepared two common types of aLC columns: an affinity column based on hybridization with immobilized nucleic acid (8 kDa) and an affinity column with immobilized mAb (150 kDa). The columns were prepared in duplicate with comparable bonding capacity (see Figure S1). An additional batch of streptavidin sorbent (ODT-18–20) was prepared and tested. The results in Supporting Information Figure S2 show that four columns packed with this batch of sorbent have a 25 nmol bonding capacity for biotin irrespective of the loading method, which consists either of a 1 nmol biotin injection or a first 15 nmol biotin injection followed by additional incremental 1 nmol injections (Figure S2).

dT25 and anti-insulin mAb columns were used for method development, calibration curve preparation, and carryover tests for approximately 100 analytical cycles. No loss of sample recovery or decrease in analyte retention time was observed during that time. The columns were stored between experiments in binding buffer at room temperature. The dT25 column was retested after 8 months of storage in a water solvent at 10 °C. More than 400 sample injections in buffer and 300 injections of in vitro transcription media were performed (Supporting Information Figure S13). No changes were observed in mRNA sample recovery, retention time, or column operating pressure.

The rationale for the development of an efficient analytical-scale affinity LC column packed with small sorbent (2.1 × 20 mm, 3.5 μm) compatible with high pressures was driven by the goal to hyphenate aLC columns with a second LC dimension. The aLC technique typically resolves the sample into two fractions (unbound and bound fractions, Figure 4A). To improve the resolution capability of aLC, we combined it with second LC separation dimensions. Figure 4 illustrates the analysis of mRNA with hyphenated dT25 × SEC chromatographic columns. The dT25 column’s unretained flow through fraction containing tailless mRNA species is directly resolved on the SEC column (SEC mobile phase is the same as the binding buffer in dT25 affinity chromatography). After the first fraction’s SEC analysis is completed (red chromatogram in Figure 4B), the polyA tail-containing mRNA fraction is eluted from the dT25 affinity column by the injection of 25 μL of water. The bound mRNA fraction is released from the dT25 column as a sharp peak permitting efficient SEC analysis (blue chromatogram in Figure 4B). SEC equilibrium was minimally disrupted by the injected water plug. The overlay of SEC chromatograms in Figure 4 confirms that the first fraction consists of shorter mRNA species, as expected for tailless mRNA or truncated mRNA species. This fraction does not contain appreciable amounts of intact polyA-tailed mRNA (no sample breakthrough in the dT25 loading step). The second fraction contains polyA-tailed mRNA at the expected quantity and its aggregates (early eluting peaks). Both the dT25 method (Figure 4A) and the dT25 × SEC method (Figure 4B) measured a consistent quantity of tailless mRNA (1.5%) in the mRNA sample. The dT25 × SEC method is preferred for the analysis of process mRNA samples, where the process media components (nucleotides, enzymes, buffers) coelute with dT25 unretained species and obscure the tailless mRNA quantitation by the dT25 method (Figure 4A).

**Figure 4:**
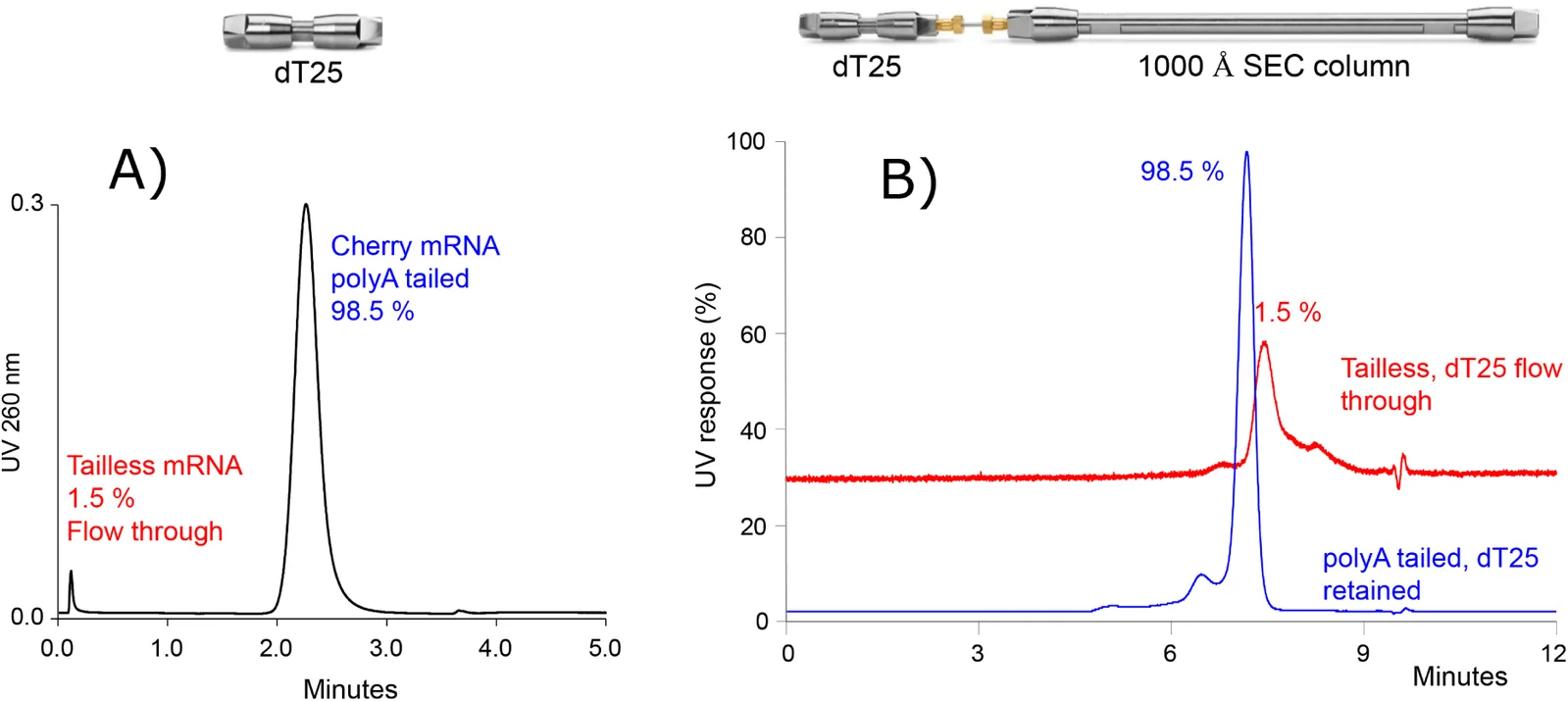
Comparison of mRNA analysis with dT25 column versus the dT25 × SEC tandem columns method. 2.5 μg of purified Cherry mRNA sample (TriLink) was injected for analysis. A) dT25 column method: The flow through peak (1.5% area) represents tailless mRNA. The retained fraction (second peak, 98.5%) is mRNA with a polyA tail. For chromatographic conditions, see the Experimental Section. B) mRNA analysis with the hyphenated dT25 × SEC method: The dT25 unretained components (flow through peak) were resolved by the SEC column (red chromatogram). After 12 min, the retained polyA-tailed mRNA was eluted from the dT25 column using a 25 μL injection of water and directly analyzed by SEC (blue chromatogram). An overlay of SEC chromatograms confirms that the tailless fraction has a smaller hydrodynamic size (loss of polyA tail) than polyA-tailed mRNA. SEC analysis of the polyA tailed fraction shows aggregates; the aggregates are retained on the dT25 column, therefore they are polyA-tailed. The normalized UV area in the first and second SEC analyses is 1.5% and 98.5%, respectively, consistent with the quantitation obtained with the dT25 column alone (panel A).

Depending on the flow rate range (0.2–0.6 mL/min) and the choice of SEC column, the aLC × SEC tandem operating pressures can reach up to 10,000 psi (69 MPa). After the dT25 column was exposed to 10,000 psi, we retested the dT25 column at 0.2–0.4 mL/min (1000–2000 psi; 6.9–13.8 MPa) and did not observe any deterioration in column performance for mRNA analysis. We concluded that the dT25 column is compatible with pressures up to 10,000 psi.

## Conclusions

In this work, we illustrate that streptavidin immobilized on 3.5 μm nonporous particles packed in a 2.1 × 20 mm column can be used for high-performance aLC. A biotinylated affinity ligand can be effectively immobilized on-column in the analytical laboratories equipped with a conventional liquid chromatography system. We achieved this using the MISER method, which was efficient and reduced the waste of biotinylated ligands, typically available only in limited quantities.

We prepared and tested two affinity columns for sensitive quantitation of mRNA and insulin. The recommended analytical conditions are applicable for routine analysis with UV detection with reported lower and upper quantitation limits. The column hardware used MaxPeak high-performance surfaces developed to minimize nonspecific adsorption of biomolecules.^33^ Preliminary evaluation of dT25 and Ins mAb aLC columns showed low injection-to-injection carryover, typically less than 1%. We conclude that dT25 affinity columns represent a new platform for sensitive quantitation of mRNA (polyA-tailed). dT25 × SEC tandem enables more advanced analysis of mRNA than the dT25 column alone and can be used to quantify tailless versus polyA-tailed mRNA species. The Ins mAb column was prepared as a model to demonstrate the immobilization of a full antibody on streptavidin columns. While the column was useful for insulin quantitation, the sample bleed from the column during the bonding and wash conditions indicates weaker analyte binding to the mAb than expected. The choice of the mAb and analytical method requires further optimization.

## Supplementary Material



## Notes

Examples of Anti-insulin mAb column preparation, column loading repeatability, and bleed using UV and LC MS analysis; dT25 column capacity for dA20 oligonucleotide and mRNA; Ins mAb column capacity for insulin; dT25 column performance and stability; Waters, MaxPeak, ACQUITY, and Xevo are trademarks of Waters Corporation or its affiliates; CaptureSelect is a trademark of Life Technologies Corporation; Milli-Q is a trademark of Merck KGaA (PDF)
