# Supplementary material for: On-Column Preparation of High-Performance Affinity Chromatography Columns
Source: Anal Chem. 2026 Jul 23;98(33):24117–25. doi: 10.1021/acs.analchem.6c01884 (PMC13523552; doi:10.1021/acs.analchem.6c01884)
Supplement: Supplementary file 1 [file ac6c01884_si_001.pdf]

Supporting information

On-column preparation of high-performance affinity chromatography columns

Martin Gilar, Beatrice Muriithi, Yeliz T. Sarisozen, Philip Wuthrich, Michael Donegan, and Kevin D. Wyndham*

Separations R&D, Waters Corporation, Milford, Massachusetts 01757 – 3696, United States;

* Corresponding author: Martin Gilar – Waters Corporation, 34 Maple Street, Milford, Massachusetts 01757 – 3696, United States, martin_gilar@waters.com

Supporting Figures:

Contents

Figure S1: Anti-Insulin mAb column loading....S2

Figure S2: Streptavidin column loading capacity (biotin) and repeatability. S3

Figure S3: Anti-insulin mAb breakthrough UV spectrum S4

Figure S4: dT25 column endcapping with biotin S5

Figure S5: LC MS analysis of biotinylated Ins mAb....S6

Figure S6: LC UV and MS analysis of dT25 column bleed S7

Figure S7: LC MS analysis of streptavidin bleed from unbounded streptavidin columns....S8

Figure S8: Ins mAb column application to insulin analysis....S9

Figure S9: dT25 column capacity for dA20 oligonucleotide....S10

Figure S10: dT25 column capacity for EGFP mRNA (997 nt) S11

Figure S11: dT25 column capacity for Cas9 mRNA (4522 nt)....S12

Figure S12: Ins mAb column capacity for insulin S13

Figure S13: dT25 column performance and longevity. S14

Table S1: Evaluation of dT25 columns performance and stability. S15

Figure S1: Anti-Insulin mAb column loading

Anti-Insulin mAb loading, 6 µL/injection (0.025 nmol/injection). Mobile phase: 100 mM sodium phosphate pH 7.4, 25 °C, 0.15 mL/min, UV 280 nm, ~ 9.5 % breakthrough is presumably non-biotinylated mAb; this makes 0.046 nmol biotinylated INS mAb/injection or 1.79 nmol of immobilized mAb.

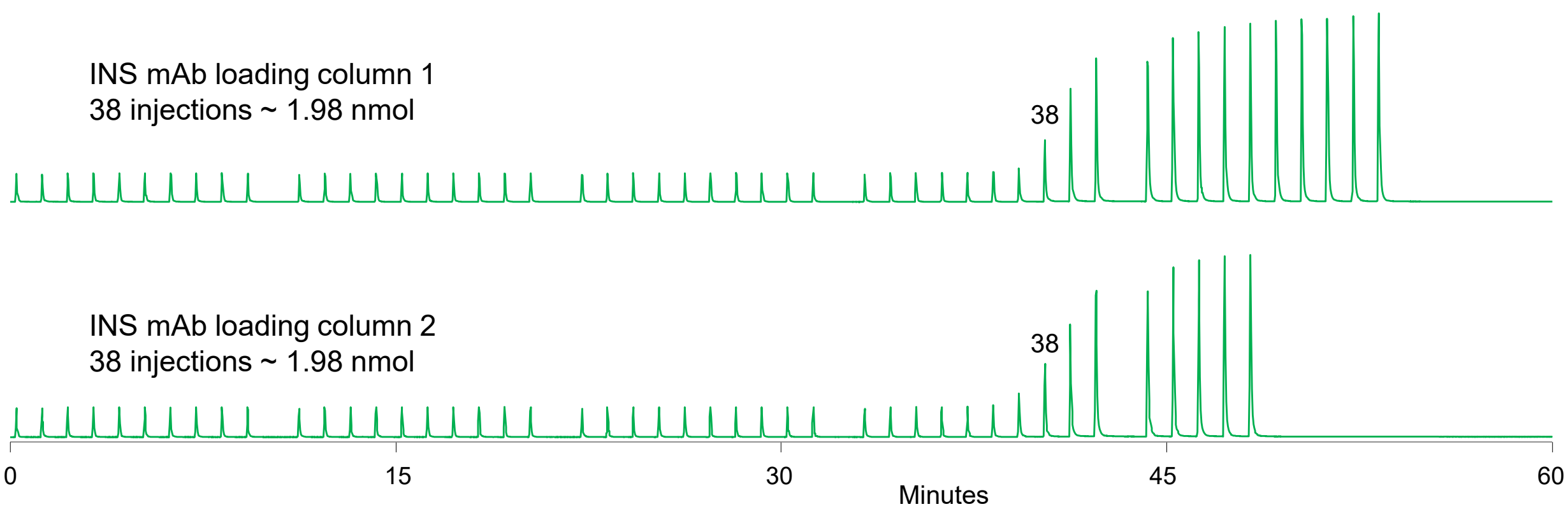

Figure S2: Streptavidin column loading capacity (biotin) and repeatability.

Three streptavidin columns (2.1 x 20 mm) were loaded with biotin, chromatograms A, B) and C), to estimate the binding capacity for free biotin. First injection (red line) was 5 mM solution of biotin in water; 3 μL injection volume corresponds to 15 nmol of biotin injected on columns. Subsequently we injected 15 injections of diluted biotin solution, each corresponding to 1 nmol biotin injection. Breakthrough of biotin (marked with arrow) occurred after total mass load of 25 nmol biotin on column. All three columns had similar loading capacity – see chromatograms A), B) and C). Chromatogram D) show the fourth column loaded with repeated 1 nmol injections of biotin. Biotin breakthrough was detected after 25 nmol biotin injections. This experiment illustrates the repeatability of column binding capacity (for biotin) the fact that column loading experiment can be shortened by loading higher concentration of analyte followed by smaller mass injections. The MISER loading method is useful in scenarios where the limited amount of biotinylated ligand is available for column preparation, and the sample must be used optimally with minimal waste in the loading experiment (loading injections can be stopped immediately after detecting breakthrough).

Chromatographic conditions: Binding buffer was 0.1 M sodium phosphate, pH 7.4, flow rate 0.15 mL/min, column temperature 25 °C, UV detection 210 nm. 50 mM biotin sample (Thermo B20656) was diluted 10 or 100-fold in water; the sample solvent was different from the running binding buffer is a source of the negative dip in the UV baseline at 210 nm, before the biotin breakthrough appears as positive signal. H-class Bio Acquity UPLC system was used for the column loading.

streptavidin sorbent batch ODT-18-20

Inj 1: 3 μL x 5 nmol (5 mM biotin solution in water) = 15 nmol of biotin loaded + Inj 2-16: 2 μL of 0.5 nmol (0.5 mM biotin solution in water) = 1 nmol of biotin per injection

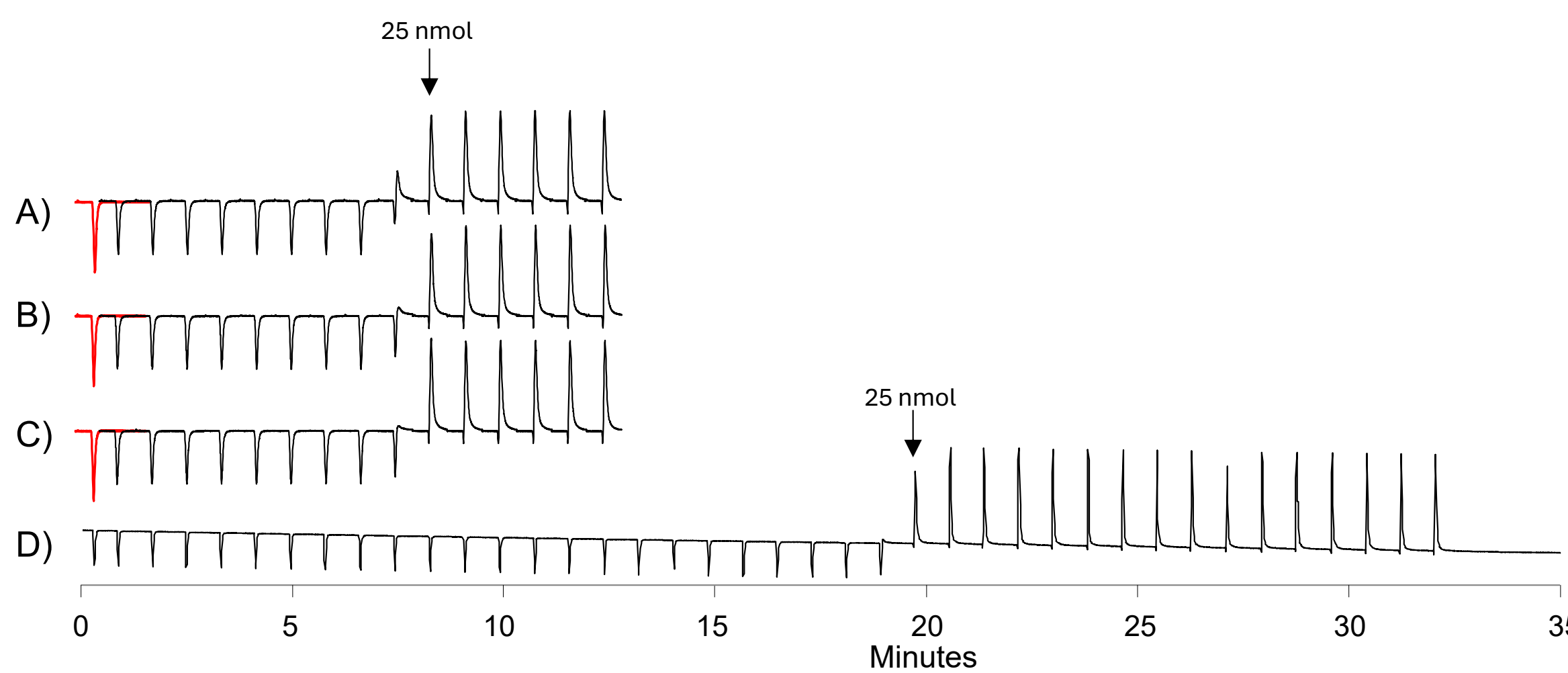

Figure S3: Anti-insulin mAb breakthrough UV spectrum

Anti-insulin mAb column preparation - breakthrough vs mAb peaks UV spectra (same experimental data as in Figure S1).

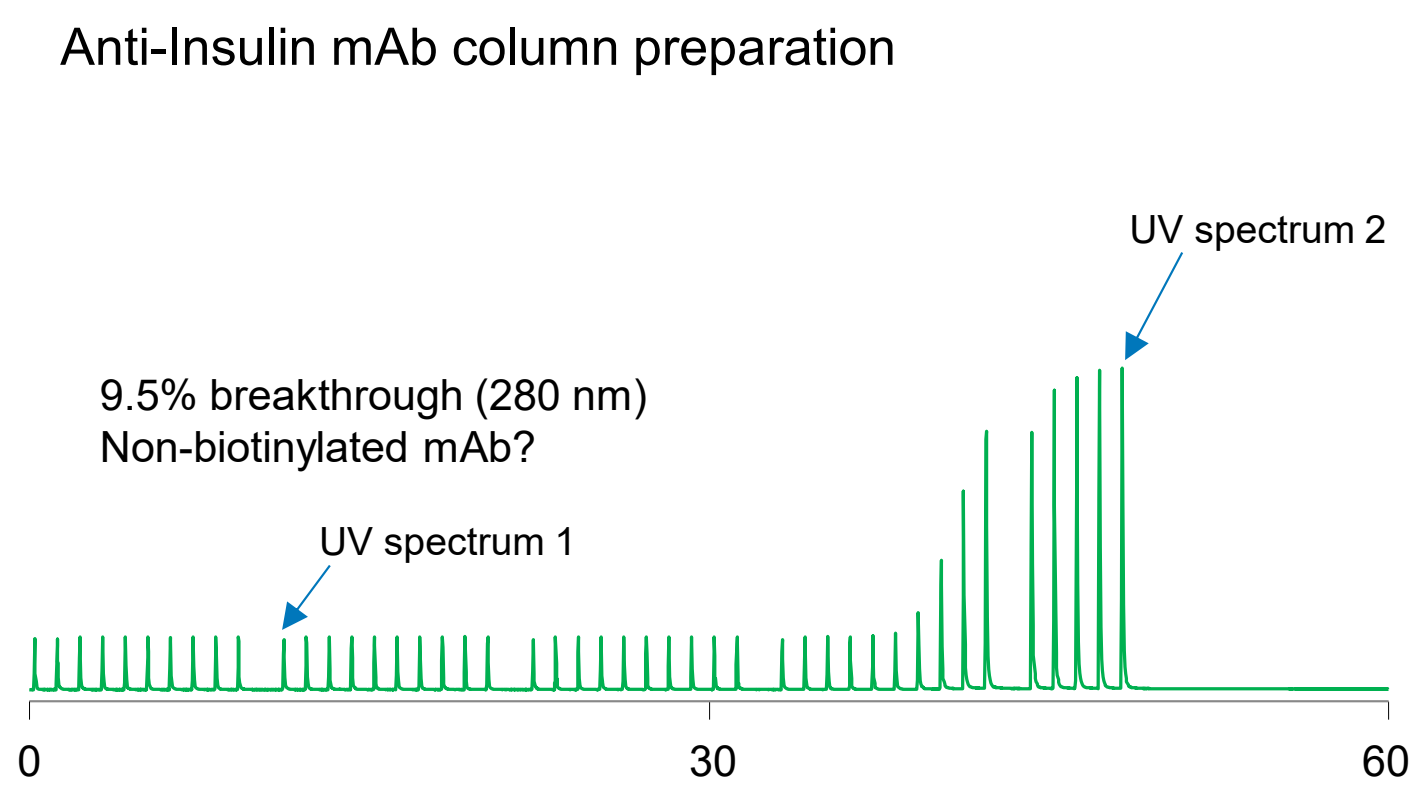

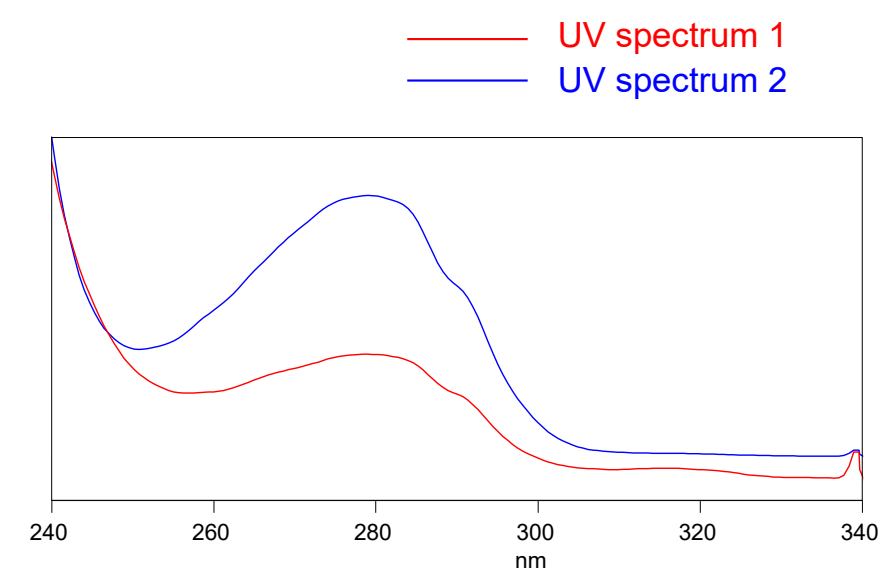

UV spectra of peaks are similar; breakthrough peak is presumably non-biotinylated version of Anti-Insulin mAb.

Figure S4: dT25 column endcapping with biotin

Endcapping of dT25 loaded streptavidin column with biotin solution. A) UV chromatogram at 210 nm indicates the breakthrough of biotin at 15th injection. B) The same experiment visualized at 260 nm indicates minor displacement of dT25 ligand during endcapping. C) UV spectra of peak 1 (near biotin breakthrough) and peak 2 (column is fully saturated with biotin). Peak 1 has characteristic spectrum of dT25, while peak 2 does not show significant bleed of dT25 ligand.

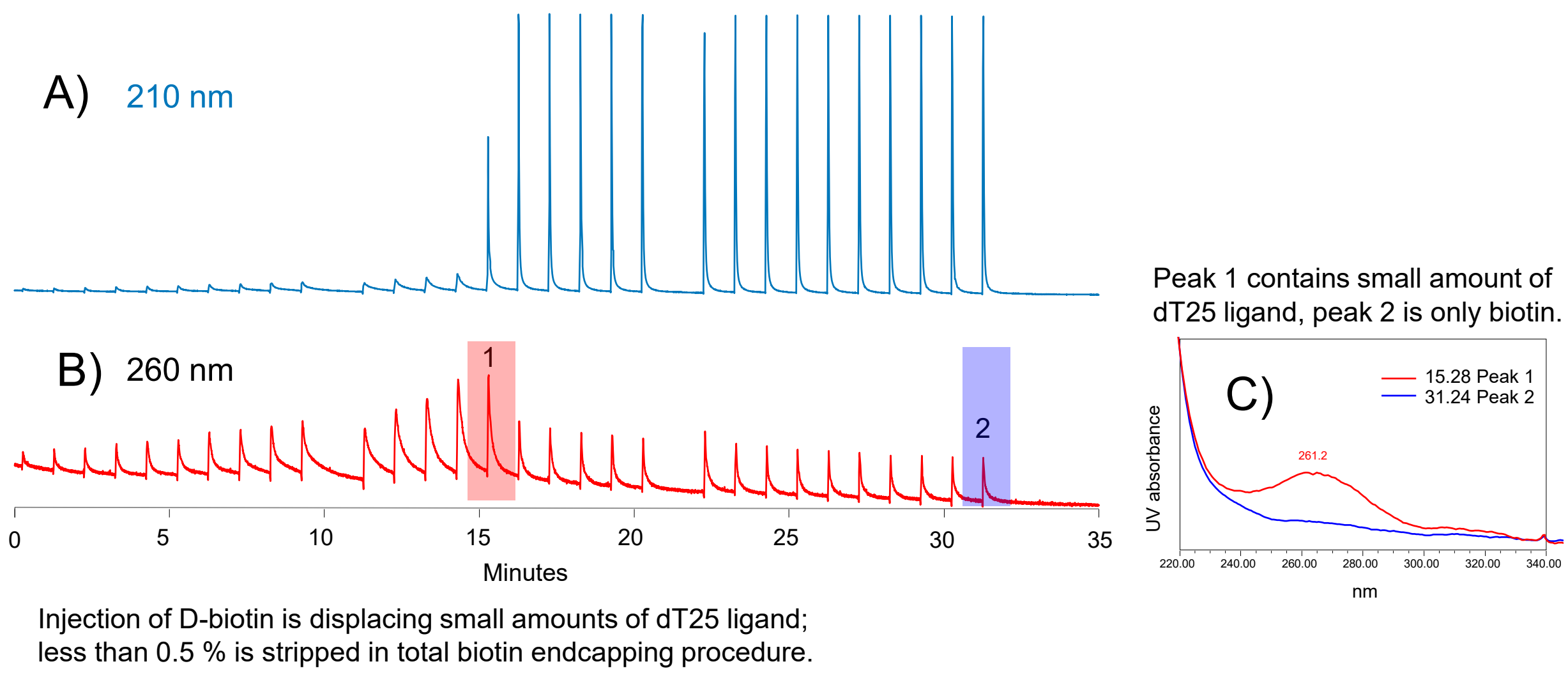

Figure S5: LC MS analysis of biotinylated Ins mAb

Light A) and heavy chain B) masses were obtained after mAb reduction and deglycosylation. Analysis of intact mAb C) was also performed after deglycosylation with PNGase F. Distribution of different biotinylated forms (0 to 5 biotin groups) was observed along with additional (unknown) mass species.

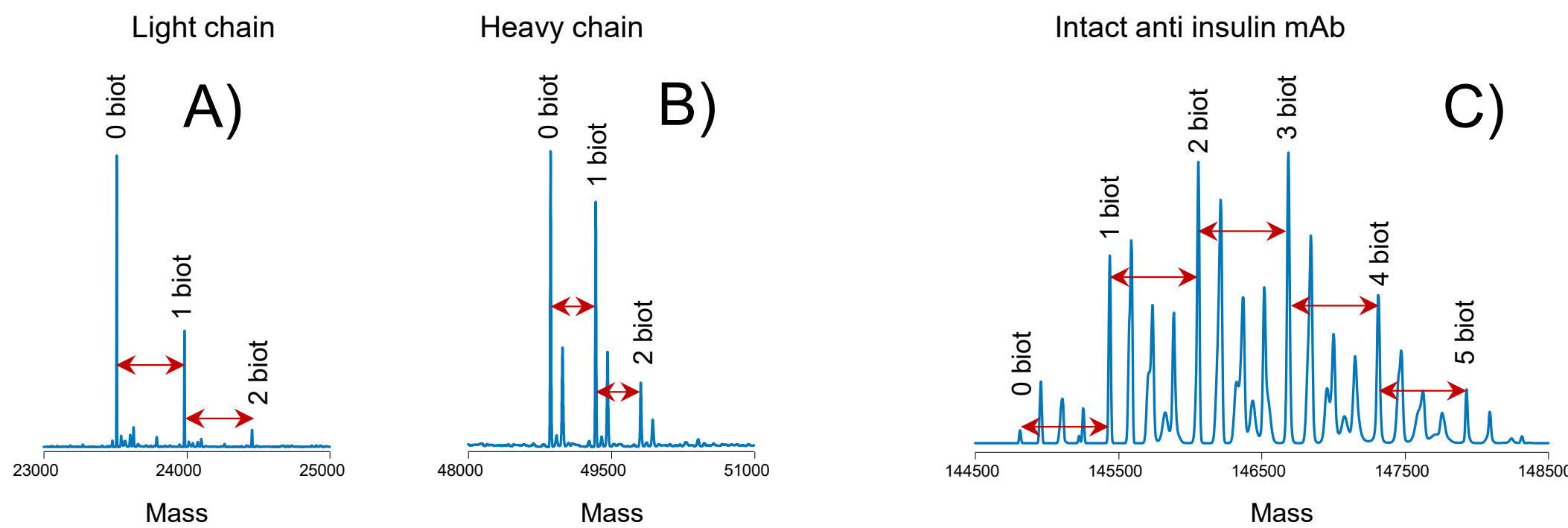

Biotin+linker mass addition is ~ 473 Da
Heavy and light chain mass was obtained after anti-insulin reduction and PNGase F de-glycosylation
Intact anti insulin mAb mass was obtained after PNGase F de-glycosylation under native conditions in 2 hr reaction. Sample preparation was performed according to New England Biolab PNGase F kit protocol. The nature of the minor peak series (Δ125 Da) in heavy chain B) and intact mAb C) is unclear.

Light chain contains 68.6% of no biotin form, 27.4% with 1biotin and 4% is doubly biotinylated
Heavy chain contains 49.0% of no biotin form, 40.1% with 1biotin and 10.9% is doubly biotinylated
Full mAb contains dominant 1-4x biotinylated forms, and lower amounts of non-biotinylated mAb and 5x biotinylated form

Figure S6: LC UV and MS analysis of dT25 column bleed

A) dT25 column was washed before use with 5 gradients. UV 260 nm background signal decreases with each subsequent elution. Red chromatogram overlayed for comparison represents the blank gradient after 25 gradients (after calibration curve experiment). Typical UV 260 nm background level is 0.002 AU. B) Column was washed with 10 mM ammonium acetate buffer, and bleed was detected with an Xevo™ G3 QTof MS instrument, negative ESI mode. MS spectrum B) was summed over ~ 0.5 min window. Deconvoluted MS spectrum from data in panel B) are shown in panel C); the deconvoluted spectrum shows both dT25 biotinylated ligand and streptavidin monomer masses.

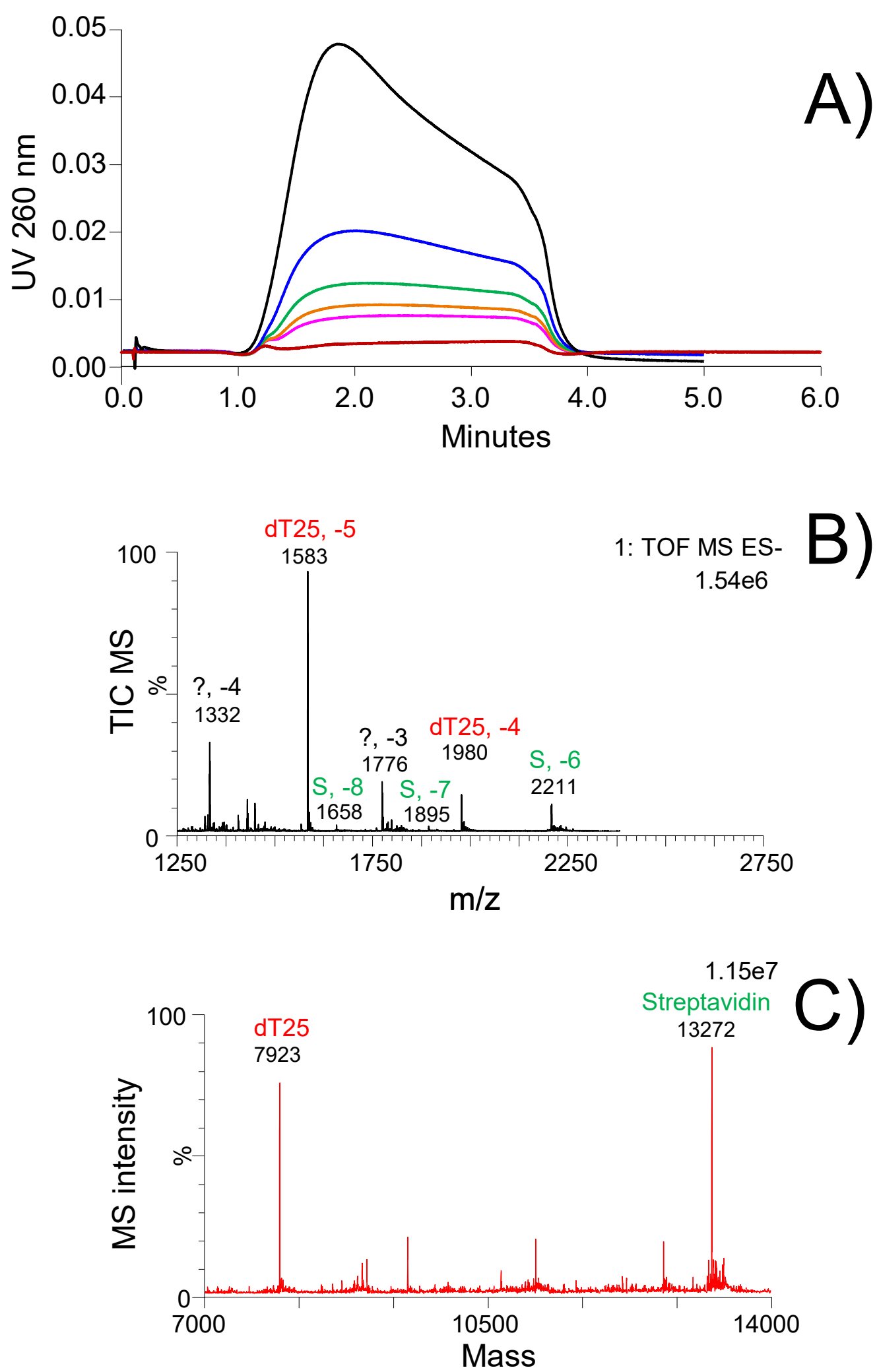

Figure S7: LC MS analysis of streptavidin bleed from unbounded streptavidin columns

A) LC MS gradient wash of unbounded streptavidin column. Conditions: 0.2 mL/min, gradient from 0.1% formic acid to 80% acetonitrile in 0.1% formic acid in 3 min. 3.6 min re-equilibration to initial conditions, Xevo G3 QTof MS, positive ESI mode. B) MS spectra summed from MS chromatogram 1.5-2 min window from data shown in panel A) show MS bleed of streptavidin monomer. C) Deconvoluted MS spectrum from data in panel B) confirm mass of streptavidin monomer MW, 13272 Da. We conclude that the column bleed consists of streptavidin and immobilized biotinylated ligand (in this case dT25 biotinylated ligand, MW 7923 Da).

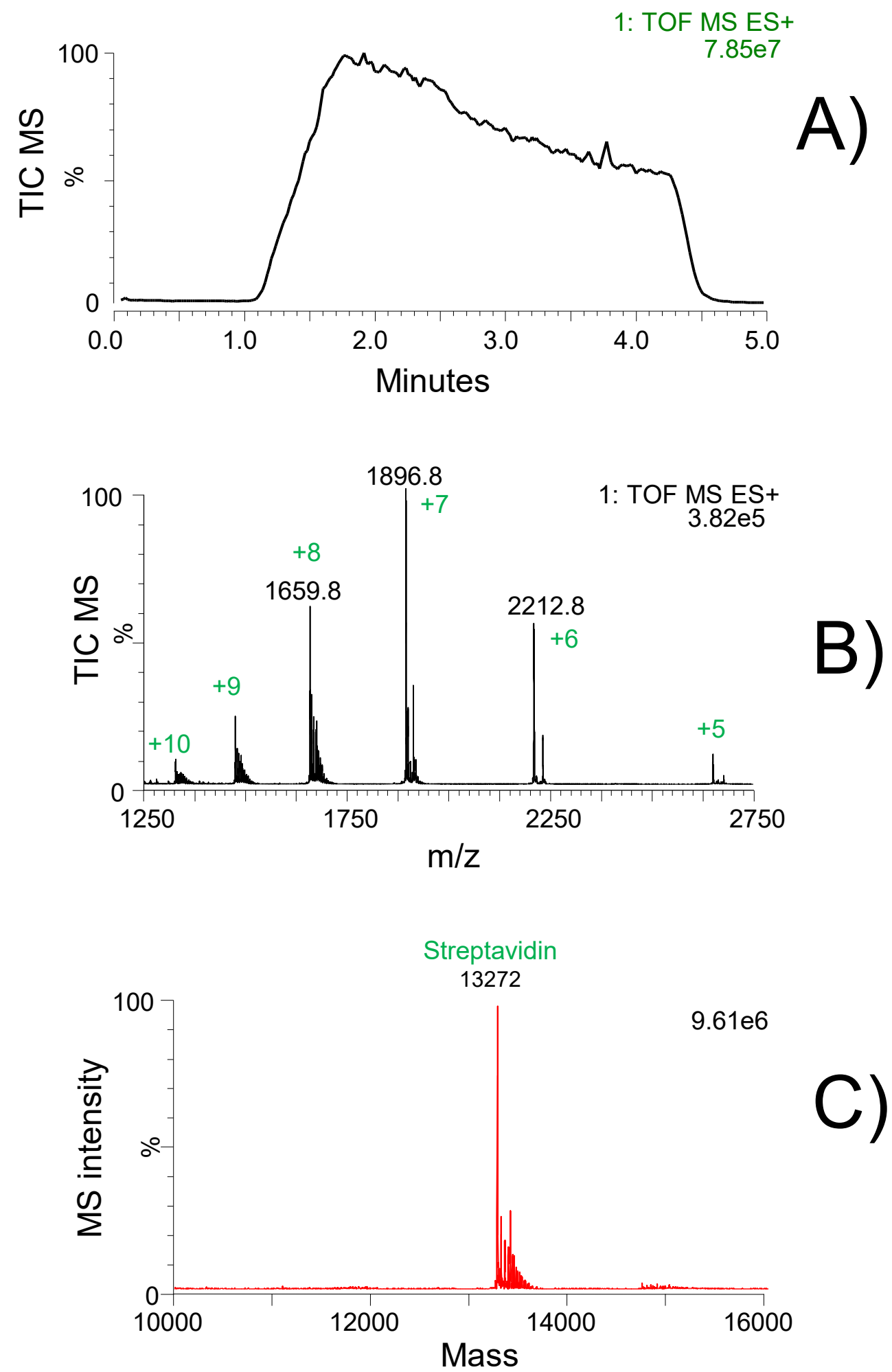

Figure S8: Ins mAb column application to insulin analysis

A) Example of insulin quantitation on affinity column. Breakthrough of insulin was observed for 10 µg injection (peak labeled insulin breakthrough). For LC conditions see Experimental section of the manuscript. B) and C) panels show calibration curve. The linearity is lost above 5 µg insulin mass load.

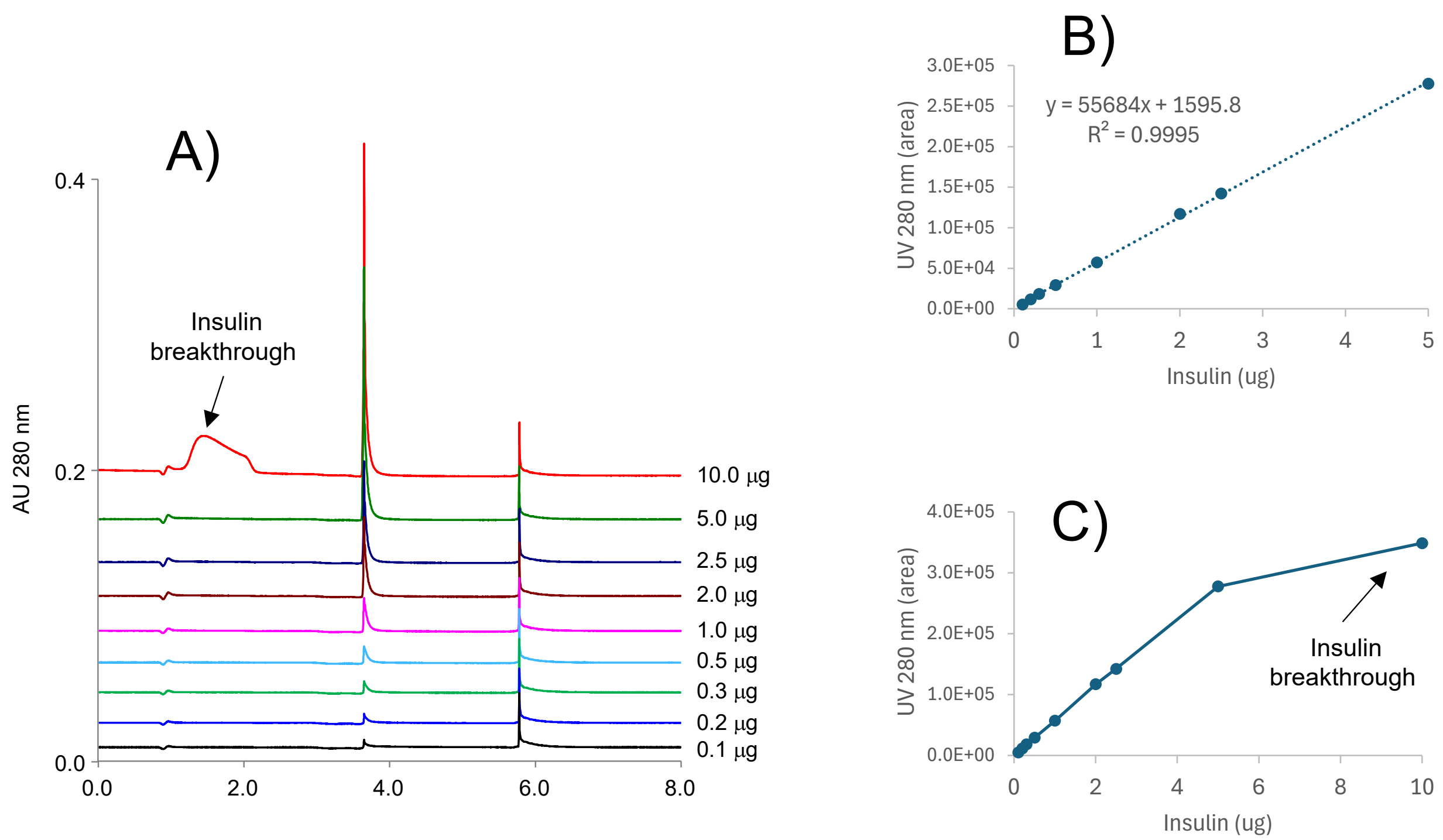

Figure S9: dT25 column capacity for dA20 oligonucleotide

MISER experiment was used to load 0.16 nmol/μL dA20 oligonucleotide sample in 3 μL injections. The breakthrough was observed at 22nd injection. Calculated dT25 column capacity for dA20 oligonucleotide was 9.62 nmol. LC conditions: flow rate 0.15 mL/min of 0.1 M sodium phosphate buffer, 25 °C, UV 260 nm. Sample injections were performed every 60 seconds; a gap of 1 min was inserted after a 10-injection series.

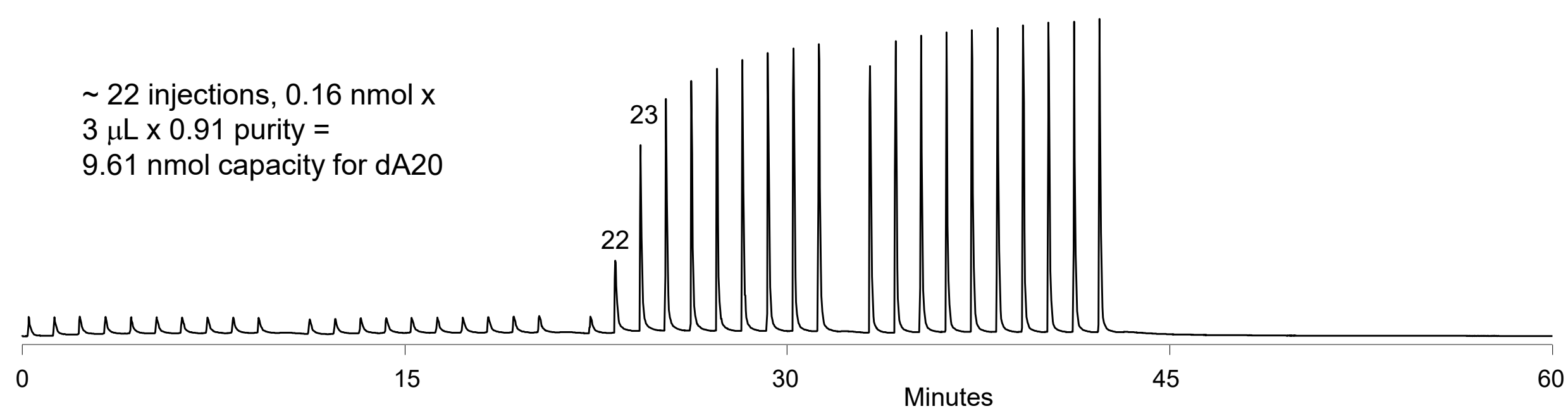

Figure S10: dT25 column capacity for EGFP mRNA (997 nt)

MISER experiment was utilized to load 1 µL injections of 0.95 mg/mL solution of EGFP mRNA (2.93 pmol/µL). The >50% breakthrough was observed at 87th injection. Calculated dT25 column capacity for EGFP mRNA was 0.251 nmol. LC conditions: flow rate 0.15 mL/min of 0.1 M sodium phosphate buffer, 25 °C, UV 260 nm. Sample injections were performed every 60 seconds; a gap of 1 min was inserted after a 10-injection series.

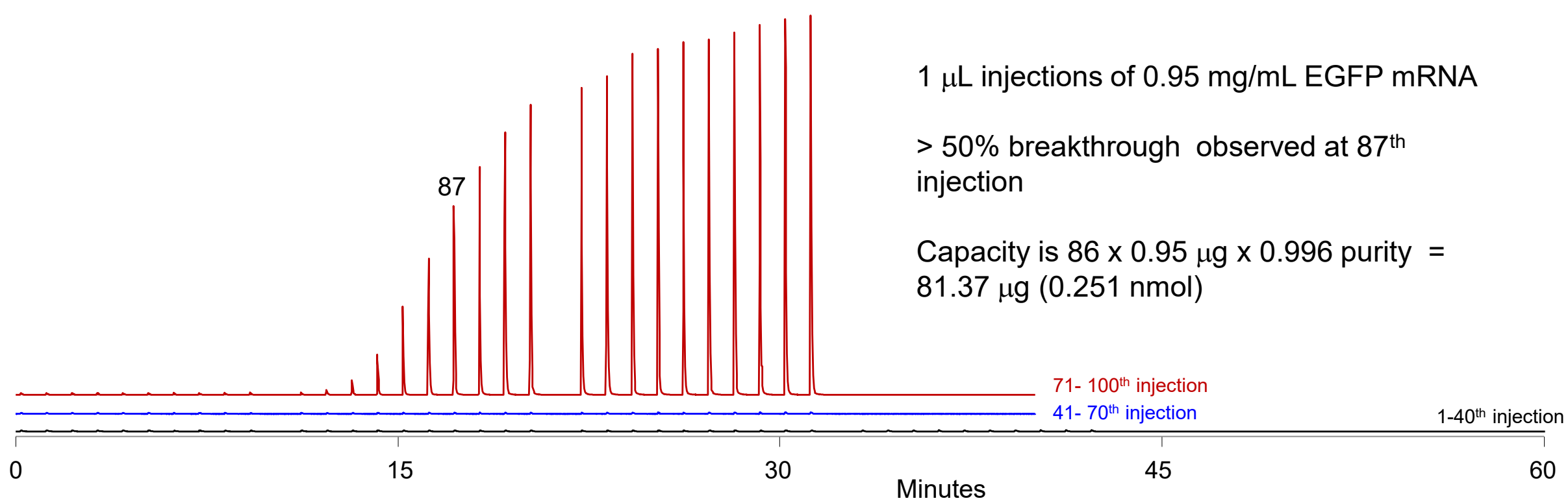

Figure S11: dT25 column capacity for Cas9 mRNA (4522 nt)

MISER experiment was utilized to load 1 µL injections of 0.95 mg/mL solution of Cas9 mRNA (0.64 pmol/µL). The >50% breakthrough was observed at 53rd injection. Calculated dT25 column capacity for Cas9 mRNA was 0.031 nmol. LC conditions: flow rate 0.15 mL/min of 0.1 M sodium phosphate buffer, 25 ?C, UV 260 nm. Sample injections were performed every 60 seconds; a gap of 1 min was inserted after a 10-injection series.

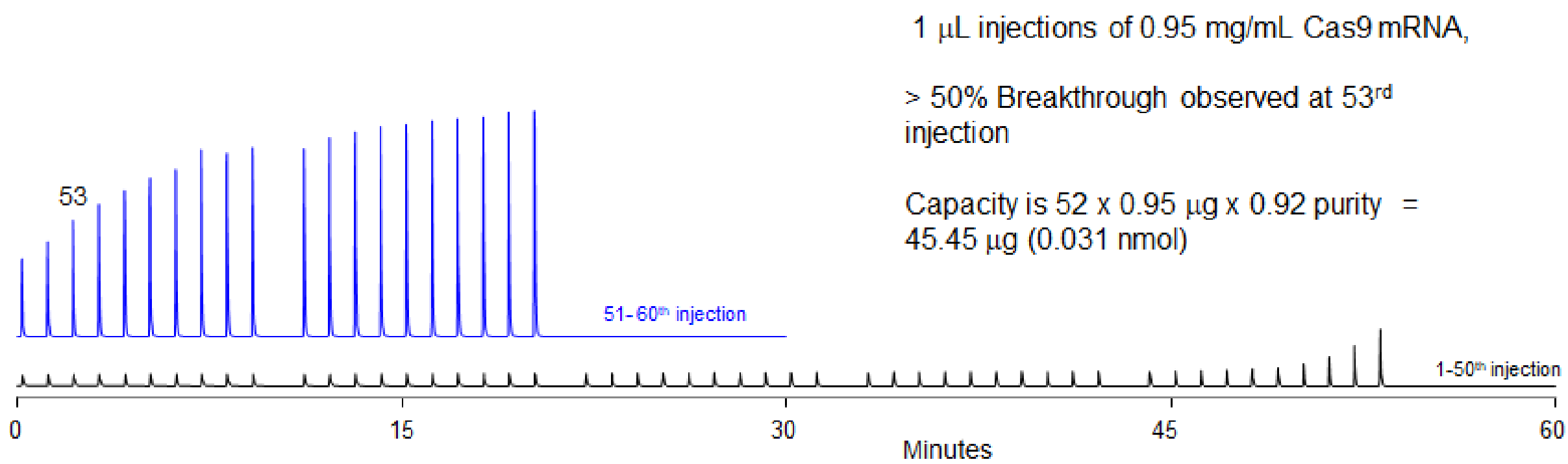

Figure S12: Ins mAb column capacity for insulin

MISER experiment was utilized to load 10 μL injections of 0.1 mg/mL solution of insulin (0.172 nmol of insulin per injection). The breakthrough was observed at 7th injection. Calculated Ins mAb column capacity was 6 μg or 1.03 nmol of insulin. LC conditions: flow rate 0.15 mL/min of 0.1 M sodium phosphate buffer, 25 °C, UV 280 nm. Insulin injections were performed every 2.5 min intervals; a gap of 1 min was inserted after 10 injections series.

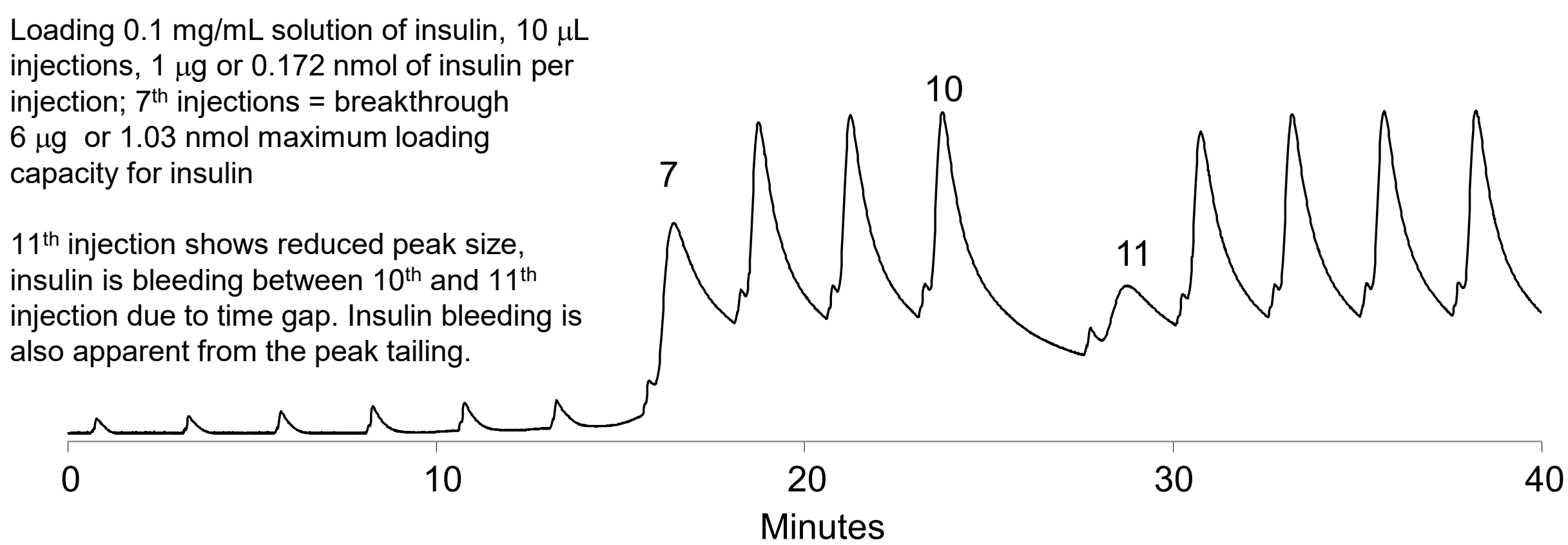

Figure S13: dT25 column performance and longevity.

A) Repeatability of 0.5 µg EPO mRNA quantitation on dT25 affinity column (prototype YTS-8-180-2; 2.1 x 20 mm column). Eight consecutive injections were performed, n=8. RSD value for the peak area was 0.41%. B) After column wash, blank injections, repeatability measurements and calibration (first 250 sample injections) the column was further exposed to 400 injections of buffer and 300 injections of in-vitro transcription medium (containing T7 RNA polymerase, NTPs, PBS buffer). After 19 blank or media injections one 0.5 µg EPO mRNA standard injection was performed and quantified. The recovery of mRNA was within the range of 97-102 % with RSD = 1.13% for 700 injections. The mRNA retention and recovery were not affected by column aging. C) The same experiment as in B), but retention time of mRNA peak is reported. Overall retention time RSD was 1.98 % with no retention time trend detected, indicating that the column has good stability at the experimental conditions. No change in column operation pressure was detected in the experiment (data not shown).

Chromatographic conditions: Mobile phase A: 0.1 M sodium phosphate, pH 7.4; mobile phase B: 10% acetonitrile, flow rate 0.4 mL/min, column temperature 25 °C, UV 260 nm, 5 mm Ti PDA cell. Gradient from 100% A to 100% B at 0 min, hold at 100% B till 2.5 min, 2.51 min equilibrate at 100% A till 5 min.

A)

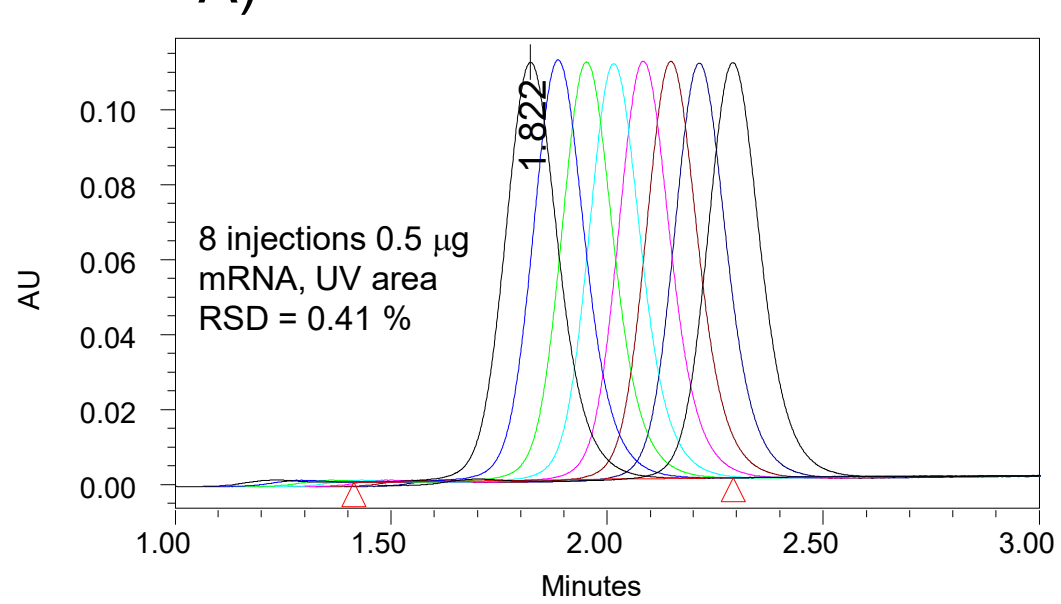

B)

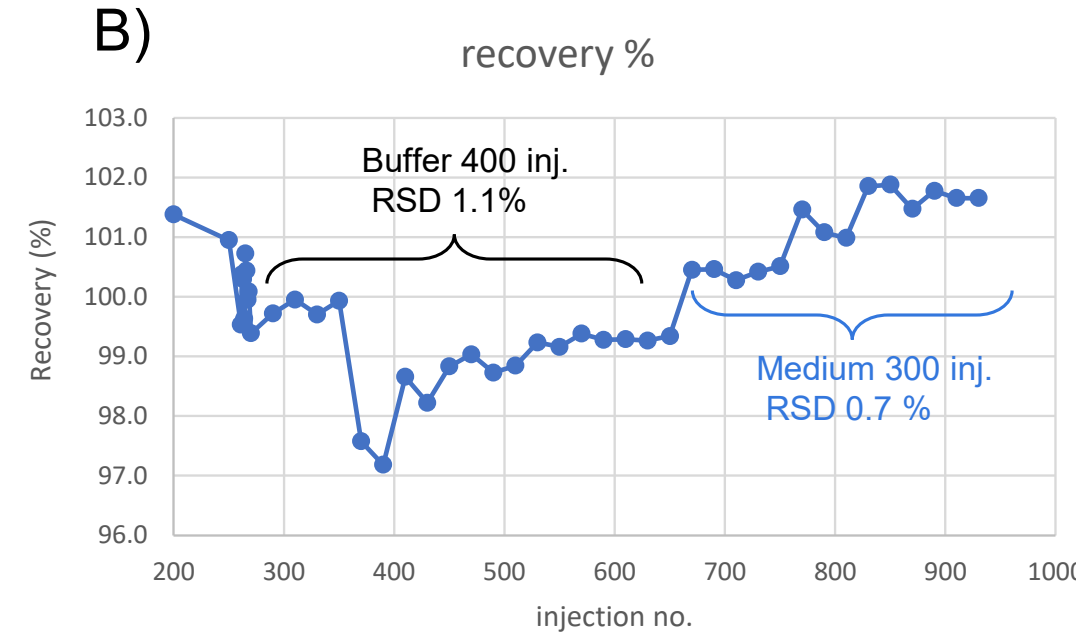

Injections 1-250: column wash, calibration, injections repeatability
Injections 250-650: buffer, every 20th injection 0.5 µg mRNA; area RSD = 1.1 %
Injections 650-950: medium, every 20th injection 0.5 µg RNA; area RSD = 0.7 %
Overall RSD = 1.13%

C)

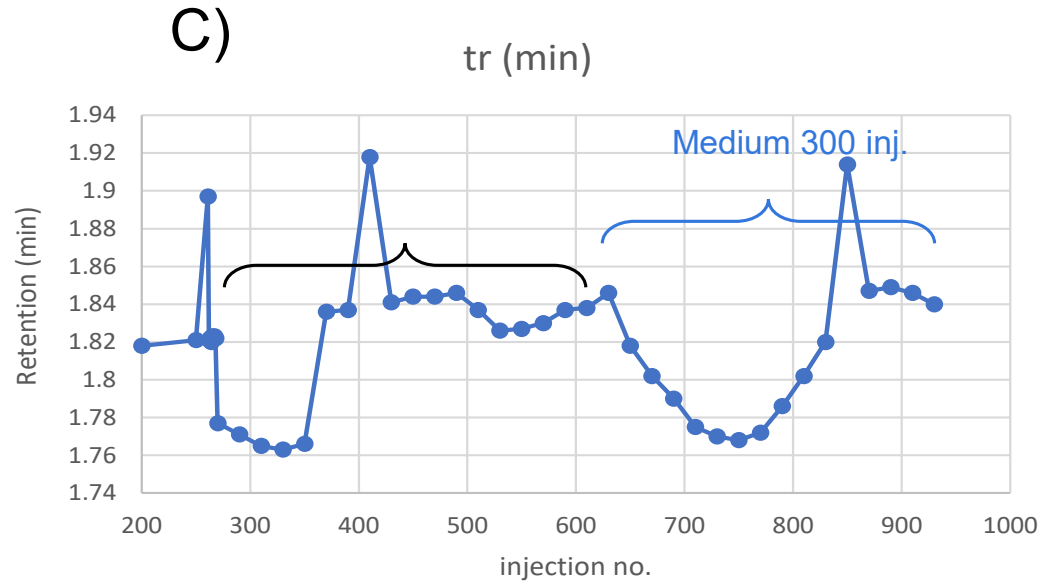

mRNA retention time repeatability between 200-950th injections, 0.5 µg mRNA standard was injected every 20th injection + repeatability experiment (44 data points), RSD = 1.98%

Table S1: Evaluation of dT25 columns performance and stability.

Column	Calibration linearity [a] R^2	mRNA recovery (%)	Carryover (%)	Observed pressure [b] (psi)
dT25 new	0.9996	98-101	< 1.0	1950
dT25, after 950 injections	0.9994	101-102	< 0.3	2000
dT25, after 8 months [c]	0.9997	98-101	< 0.2	1970

[a] mRNA calibration range 0-5 μg injected on column.

[b] Pressure was monitored by H-class Bio ACQUITY UPLC system, ~ 600 psi is due to system tubing (at 0.4 mL/min).

[c] dT25 column was stored between day-to-day experiments at 8 °C in running buffer or water. For long-term storage 10% acetonitrile was used.
